# Supplementary material for: Determinants of Directionality and Efficiency of the ATP Synthase Fo Motor at Atomic Resolution
Source: J Phys Chem Lett. 2022 Jan 5;13(1):387–92. doi: 10.1021/acs.jpclett.1c03358 (PMC8762653; doi:10.1021/acs.jpclett.1c03358)
Supplement: Supplementary file 1 — jz1c03358_si_001.pdf [file jz1c03358_si_001.pdf]

Supporting Information for: Determinants of Directionality and Efficiency of the ATP Synthase F_o Motor at Atomic Resolution

Antoni Marciniak,† Pawel Chodnicki,† Kazi A Hossain,† Joanna Slabonska,† and Jacek Czub*,†,‡

†*Department of Physical Chemistry, Gdansk University of Technology, Gdansk, Poland*

‡*BioTechMed Center, Gdansk University of Technology, Gdansk, Poland*

E-mail: jacek.czub@pg.edu.pl

Results and discussion

To more thoroughly analyze the protonation state of F_o for the purpose of our free energy simulations, in addition to computing pKa values of the B and R site glutamates (8.4±0.3 and 1.9±0.2, respectively), we also determined pKa's for all of the remaining proton-carrying carboxylates of the c-ring and the two glutamate residues located in the proton-binding and proton-release half-channels (E223 and E162, respectively). As expected from their location in the lipid environment (see Fig. S3), we found all c-ring carboxylates apart from these at the B and R sites to be strongly basic, with pKa values of at least 11, which correlates well with the open and closed conformations the carboxylates are known to adopt (see Fig S20).[1] At the same time, the pKa of E223 in the proton-binding half-channel is shifted by 1.4 units with respect to the bulk solvent (to 5.5±0.2). As the difference between B site carboxylate' pKa and E223's pKa remains relatively high (2.5 units), we believe that shift in the pKa of

E223 is an indication of proton affinity increase with respect to the decrease in distance to the c-ring. Moreover, we found that when E223 is mutated to alanine (E223A) water molecules are almost entirely excluded from the vicinity of the B site carboxylate and consequently its pKa rises sharply to 14.7±0.1 . Therefore, we propose that the role of E223 is largely to facilitate access of water to the c-ring binding site on the perimitochondirial side. This finding also supports the notion that the difference in pKa between the B and R site carboxylates arises from the strikingly different degree of hydration (see Fig S8,S7). For E162 we found pKa to be shifted considerably (by 1.9±0.2 units) to 6.0. Difference of 4 units between R site carboxylate and E162 suggests that E162 may participate in shuttling protons released into the half-channel from the c-ring R site.

We were also interested in the effect of the central (R176) and secondary (R169) arginine residues on the proton affinity of the R site carboxylate. As described in the main text, by comparing pKa of the wild type and R176K mutant with the R176A mutant, we found that both positively charged residues, arginine and lysine, stabilize the deprotonated state of the carboxylate to a similar extent, lowering its pKa by ∼5 units from a value of 6.9±0.1 calculated for the R176A mutant to 1.9±0.2 (WT) and 2.2±0.4 (R176K). In contrast, the secondary arginine shifts the pKa of the R site only by ∼1 unit, as the pKa of the R169A mutant was computed to be 2.8±0.2. Since the effect of R169 on the protonation behavior of the R site carboxylate is weak and the rotation free energy profile of the R169A mutant remains asymmetric around 0° (Fig. S16), we conclude that the presence of the secondary arginine in the yeast F_o does not qualitatively affects the general rotary mechanism.

Methods

System preparation

The cryo-EM structure (PDB Id: 6B2Z)[2] of yeast mitochondrial ATP synthase (isolated F_o monomer) was taken as an initial configuration. As e- and g-subunit, located far from the

crucial a/c-ring interface, were modeled in the cryo-EM structure as poly-Ala chains, they were completely omitted. Using the CHARMM-GUI Membrane Builder,[3–5] the protein was embedded in a lipid bilayer oriented perpendicularly with respect to the z-axis and composed of 151 POPC, 118 POPE, and 55 $TOCL^{-2}$ (tetraoleoyl cardiolipin) molecules (46.6, 36.4 and 17 mol %, respectively), reflecting the composition of the inner mitochondrial membrane in yeast.[6] The system was solvated with 25201 TIP3P water molecules in a 123.85 × 123.85 × 91.422 Å rectangular box and the number of K^+ and Cl^- ions was adjusted to maintain a physiological salt concentration of 0.15 M and neutralize the net charge of the system. One POPC and one POPE molecule per leaflet were manually inserted inside the c-ring, to seal its central channel, similarly to the previous treatment.[7]

System was subjected to two-step minimization (first keeping all protein atoms fixed and then without any constraints) followed by preliminary relaxation of the protein using CHARMM-GUI protocol (short MD runs with progressively weaker restraints on protein atoms). Next, the system was simulated with the protein backbone atoms harmonically restrained to their initial positions with a force-constant of $1000\,\mathrm{kJ/(mol{\cdot}nm^2)}$ for 500 ns, and only then equilibrated without restraints for another 700 ns. Random frames from the last 100 ns of this restraint-free run were picked as initial coordinates for five independent unbiased MD simulations of the wild type F_o, each lasting 1 μs. The mutants of F_o, R176A, R176K, R169A and E223A, were prepared by substituting R176, R169 or E223 residues with alanine or lysine, and subject to 2 μs of unbiased MD simulations, using random frames from the WT equilibrium trajectory as initial coordinates. In the systems where changes in protonation states or mutations created non-zero net charge, the number of ions in aqueous phase was adjusted to neutralize the system.

Simulation parameters

All molecular dynamics (MD) simulations were performed using GROMACS[8] and the CHARMM36m force field.[9] The simulations were carried out in the isothermal-isobaric

(NPT) ensemble using periodic boundary conditions in 3D. The constant temperature was kept at 310 K using the Nose-Hoover thermostat[10] and the pressure was maintained at 1 bar semi-isotropically (separately in the plane of the bilayer and perpendicular to the bilayer) using Parrinello-Rahman algorithm.[11] Long-range electrostatic interactions were evaluated using the Particle Mesh Ewald (PME)[12] method with a real-space cut-off of 1.2 nm. Van der Waals interactions were evaluated using a smooth cut-off of 1.2 nm with a switching distance of 1 nm. Bond lengths were constrained using the SHAKE[13] (for water) or P-LINCS[14] (for protein and lipids) algorithm. The equations of motion were integrated using a leap-frog algorithm with a time step of 2 fs.

$\mathbf{pK_a}$ calculations

To determine pKa values of the c-ring carboxylates as well as two glutamate residues located in the proton-binding and proton-release half-channels (E223 and E162, respectively), we used alchemical free energy calculations. To this end, a free glutamic acid molecule capped with N-methyl (NME) and acetyl (ACE) termini was added to the F_o system as a reference, and kept in bulk solution, at a minimal distance of 3 nm from the membrane center with the use of a one-sided harmonic potential applied to the z component of the vector connecting the centers of mass of the reference glutmate and the c-ring using PLUMED (topologies were generated using our in-house script (`https://gitlab.com/KomBioMol/proton_alchemist`).[15]

To predict the pKa shift for a given c-ring carboxylate with respect to the reference glutamate, the system was transformed, using a switching parameter λ, from the initial state with the c-ring carboxylate protonated and the reference glutamate deprotonated (state A) to the final state with the reversed protonation states (state B), and vice versa. Due to this treatment the simulated system was neutral at all λ-points and the obtained $\Delta\Delta$G values, describing the difference in the proton affinity between both environments, could be used directly to compute the pKa shift with respect to the experimental pKa in aqueous solution (4.1 for the glutamic acid side chain). The number of λ-points (or windows) was chosen to be 20

and the system was simulated in these windows using Hamiltonian replica-exchange molecular dynamics until the convergence of ΔΔG was reached (up to ∼200 ns). To optimize λ values, we used our in-house script (`https://gitlab.com/KomBioMol/converge_lambdas`) that iteratively restarts short replica-exchange runs until λ-values yielding equal exchange rates between neighboring windows (here ∼20%)) are found.[16] ΔΔG values were determined using Bennett acceptance ratio,[17] as implemented in the Gromacs package and the pK_a shifts were then calculated according to the formula $\Delta pK_a = \log_{10}\left(\exp^{\frac{-\Delta\Delta G}{RT}}\right)$

Free energy profiles for the c-ring rotation

To determine the changes in the free energy accompanying the rotation of the c-ring by one c-subunit in the wild-type F_o and its R176A, R176K, R169A and E223A mutants, the umbrella sampling (US) technique was applied. As a reaction coordinate, θ, we used the angle of rotation of the c-ring about the fixed vertical axis passing through its center. For each protein, to span the range of θ corresponding to the rotation by c-subunit (0–36° and −36–0° for the synthesis and hydrolysis direction, respectively) we used 13 equally-spaced US 'windows' separated by 3°. The stationary isotropic potential, as described by Kutzner et al.,[18] with a force constant of 0.5 kJ/(mol·nm^2) was used to restrain the systems in each of these windows. The initial configurations for the US windows were taken from the enforced-rotation simulations in which the c-ring was driven to rotate at a constant angular rate of 0.36 deg/ns by an externally applied isotropic potential with a force constant of 500 kJ/(mol·nm^2). To mimic the effect of the peripheral stalk connecting the F_o and F_1 portion and prevent the F_o protein to rotate as a whole, the backbone atoms of the membrane-embedded portion of b-subunit (see Fig. S2) were harmonically restrained to their initial positions with a force-constant of 1000 kJ/(mol·nm^2). Each of the US windows were simulated for 600 ns (with the exception of the R176K mutant, which was run for 700 and 800 ns in the synthesis and hydrolysis direction, respectively) and first 200 ns were omitted from analysis. Free energy profiles were determined using the multistate Bennett acceptance

ratio (MBAR) method, as implemented in pymbar.[19]

Conformational free energy profiles of Arg176

To determine the conformational preference of the guanidine moiety of R176 to locate in one of the two proton-access half-channels, we performed well-tempered metadynamics simulations[20] of the above described F_o system in all four possible protonation states of binding and release site carboxylates. The reaction coordinate was defined as the difference between two distances: i) the center-of-mass (com) distance between the guanidine moiety and the side chain of the R site glutamate (d1 in Fig. S13), and ii) the com distance between the guanidine moiety and the side chain of the glutamic acid in first c-subunit post binding site (d2 in Fig. S13). The metadynamics have been performed using the Plumed plugin.[15] Simulations were run with 5 walkers for each protonation state and the sampled range of the reaction coordinate ($d1 - d2$) was restricted to the −1.3–0.15 nm interval, using semi-harmonic restraints with a force constant of 3000 kJ/mol. Simulations were run until convergence was reached, for 81 ns per walker (405 ns in total) for R^-B^0 protonation state, 152 ns per walker (760 ns in total) for R^0B^- protonation state, 86 ns per walker (430 ns in total) for R^-B^- protonation state and 90 ns per walker (450 ns in total) for R^0B^0 protonation state. In each case, the biasing potential was updated every 1000 steps by depositing gaussian-shaped functions along the reaction coordinate with the initial height of 0.1 kJ/mol, width of 0.025 nm and a bias factor for well-tempering set to 15.

Interaction analysis

To evaluate the individual enthalpic contributions to the rotation free energies, we calculated electrostatic and van der Waals (vdW) interaction energies between the key residues present at the interface between the c-ring and a-subunit, the remaining protein residues, solvent including ions, and lipids. The binding and release site glutamates and the a-subunit residues within 1 nm from the R site carboxylate forming the interface were selected for

the residue-wise analysis (for a complete list of residues and their location at the c-ring/a interface, see Fig. S18). Using Boltzmann-reweighted umbrella sampling data, we computed the average electrostatic and vdW interaction energies between all the pairs considered as a function of the rotation angle. After adding electrostatic and vdW energies, the average slope of the resulting angle-dependence in the 0–18° range was determined by linear fitting, and was used as measure of a given pairwise contribution (Fig. S9, Fig. S10, Fig. S11 and Fig. S12). To extract contributions responsible for the F_o directionality the slopes in the hydrolysis direction were subtracted from those in the synthesis direction and shown in Fig. 3.

References

(1) Pogoryelov, D.; Krah, A.; Langer, J. D.; Yildiz, Ö.; Faraldo-Gómez, J. D.; Meier, T. Microscopic rotary mechanism of ion translocation in the F(o) complex of ATP synthases. *Nature Chemical Biology* **2010**, *6*, 891–899.

(2) Guo, H.; Bueler, S. A.; Rubinstein, J. L. Atomic model for the dimeric FO region of mitochondrial ATP synthase. *Science* **2017**, *358*, 936–940.

(3) Jo, S.; Kim, T.; Iyer, V. G.; Im, W. CHARMM-GUI: A web-based graphical user interface for CHARMM. *Journal of Computational Chemistry* **2008**, *29*, 1859–1865.

(4) Lee, J. et al. CHARMM-GUI Input Generator for NAMD, GROMACS, AMBER, OpenMM, and CHARMM/OpenMM Simulations Using the CHARMM36 Additive Force Field. *Journal of Chemical Theory and Computation* **2016**, *12*, 405–413.

(5) Wu, E. L.; Cheng, X.; Jo, S.; Rui, H.; Song, K. C.; Dávila-Contreras, E. M.; Qi, Y.; Lee, J.; Monje-Galvan, V.; Venable, R. M.; Klauda, J. B.; Im, W. CHARMM-GUI Membrane Builder toward realistic biological membrane simulations. *Journal of Computational Chemistry* **2014**, *35*, 1997–2004.

(6) Zinser, E.; Sperka-Gottlieb, C.; Fasch, E.-V.; Kohlwein, S. D.; Paltauf, F.; Daum, G. Phospholipid Synthesis and Lipid Composition of Subcellular Membranes in the Unicellular Eukaryote Saccharomyces cerevisiae. *Journal of Bacteriology* **1991**, *173*, 2026–2034.

(7) Zhou, W.; Marinelli, F.; Nief, C.; Faraldo-Gómez, J. D. Atomistic simulations indicate the c-subunit ring of the F1Fo ATP synthase is not the mitochondrial permeability transition pore. *eLife* **2017**, *6*.

(8) Van Der Spoel, D.; Lindahl, E.; Hess, B.; Groenhof, G.; Mark, A. E.; Berendsen, H.

J. C. GROMACS: Fast, flexible, and free. *Journal of Computational Chemistry* **2005**, *26*, 1701–1718.

(9) Huang, J.; Rauscher, S.; Nawrocki, G.; Ran, T.; Feig, M.; de Groot, B. L.; Grubmüller, H.; MacKerell, A. D. CHARMM36m: an improved force field for folded and intrinsically disordered proteins. *Nature Methods* **2017**, *14*, 71–73.

(10) Nosé, S. A unified formulation of the constant temperature molecular dynamics methods. *The Journal of Chemical Physics* **1984**, *81*, 511–519.

(11) Parrinello, M.; Rahman, A. Polymorphic transitions in single crystals: A new molecular dynamics method. *Journal of Applied Physics* **1981**, *52*, 7182–7190.

(12) Essmann, U.; Perera, L.; Berkowitz, M. L.; Darden, T.; Lee, H.; Pedersen, L. G. A smooth particle mesh Ewald method. *The Journal of Chemical Physics* **1995**, *103*, 8577–8593.

(13) Ryckaert, J.-P.; Ciccotti, G.; Berendsen, H. J. C. Numerical integration of the cartesian equations of motion of a system with constraints: molecular dynamics of n-alkanes. *Journal of Computational Physics* **1977**, *23*, 327–341.

(14) Hess, B. P-LINCS: A Parallel Linear Constraint Solver for Molecular Simulation. *Journal of Chemical Theory and Computation* **2008**, *4*, 116–122.

(15) Tribello, G. A.; Bonomi, M.; Branduardi, D.; Camilloni, C.; Bussi, G. PLUMED 2: New feathers for an old bird. *Computer Physics Communications* **2014**, *185*, 604–613.

(16) Wieczór, M.; Czub, J. Telomere uncapping by common oxidative guanine lesions: Insights from atomistic models. *Free Radical Biology and Medicine* **2020**, *148*, 162–169.

(17) Bennett, C. H. Efficient estimation of free energy differences from Monte Carlo data. *Journal of Computational Physics* **1976**, *22*, 245–268.

(18) Kutzner, C.; Czub, J.; Grubmüller, H. Keep It Flexible: Driving Macromolecular Rotary Motions in Atomistic Simulations with GROMACS. *Journal of Chemical Theory and Computation* **2011**, *7*, 1381–1393.

(19) Shirts, M. R.; Chodera, J. D. Statistically optimal analysis of samples from multiple equilibrium states. *The Journal of chemical physics* **2008**, *129*, 124105.

(20) Barducci, A.; Bussi, G.; Parrinello, M. Well-Tempered Metadynamics: A Smoothly Converging and Tunable Free-Energy Method. *Phys. Rev. Lett.* **2008**, *100*, 020603.

Table S1: Range of residues included in the simulation for each of the simulated subunits of the F_o complex

Subunit	range
8	8–48
a	27–249
d	240–283
f	32–86
j	1–36
k	5–28
b	8–103
c (×10)	1–75

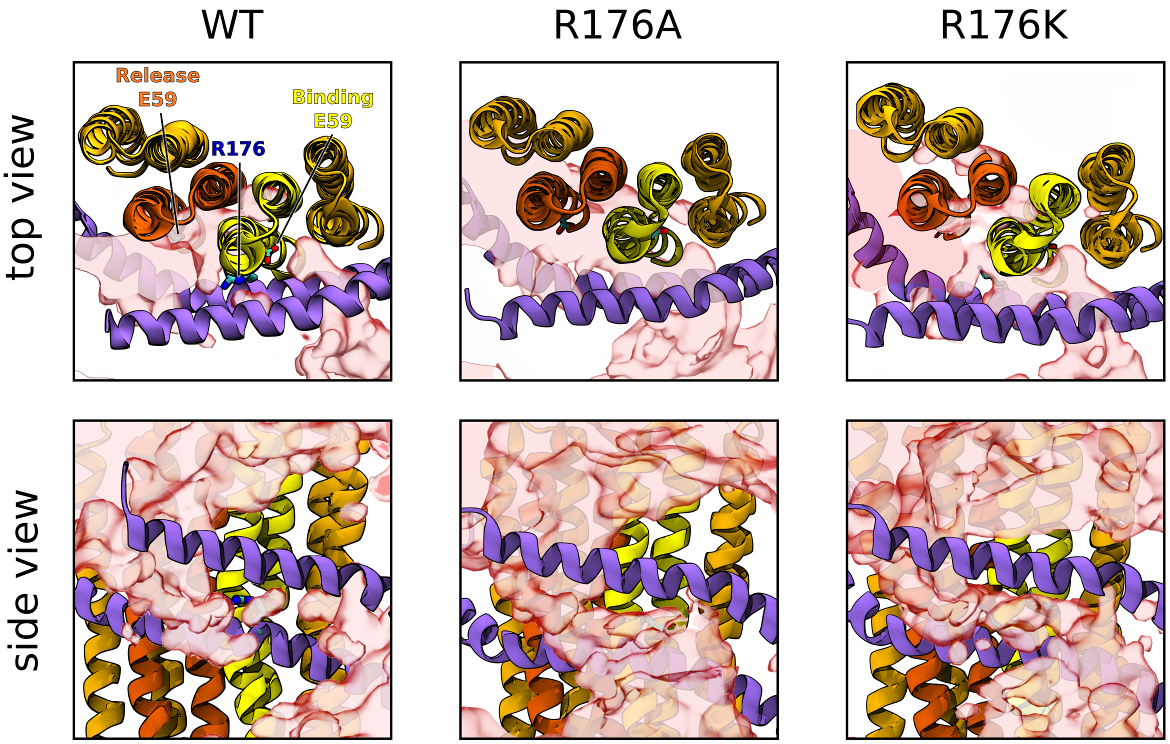

Figure S1: Top and side view of the average water densities (transparent red surfaces) at the interface between a-subunit and the c-ring in the wild type F_o protein, as well as in its R176A and R176K mutants.

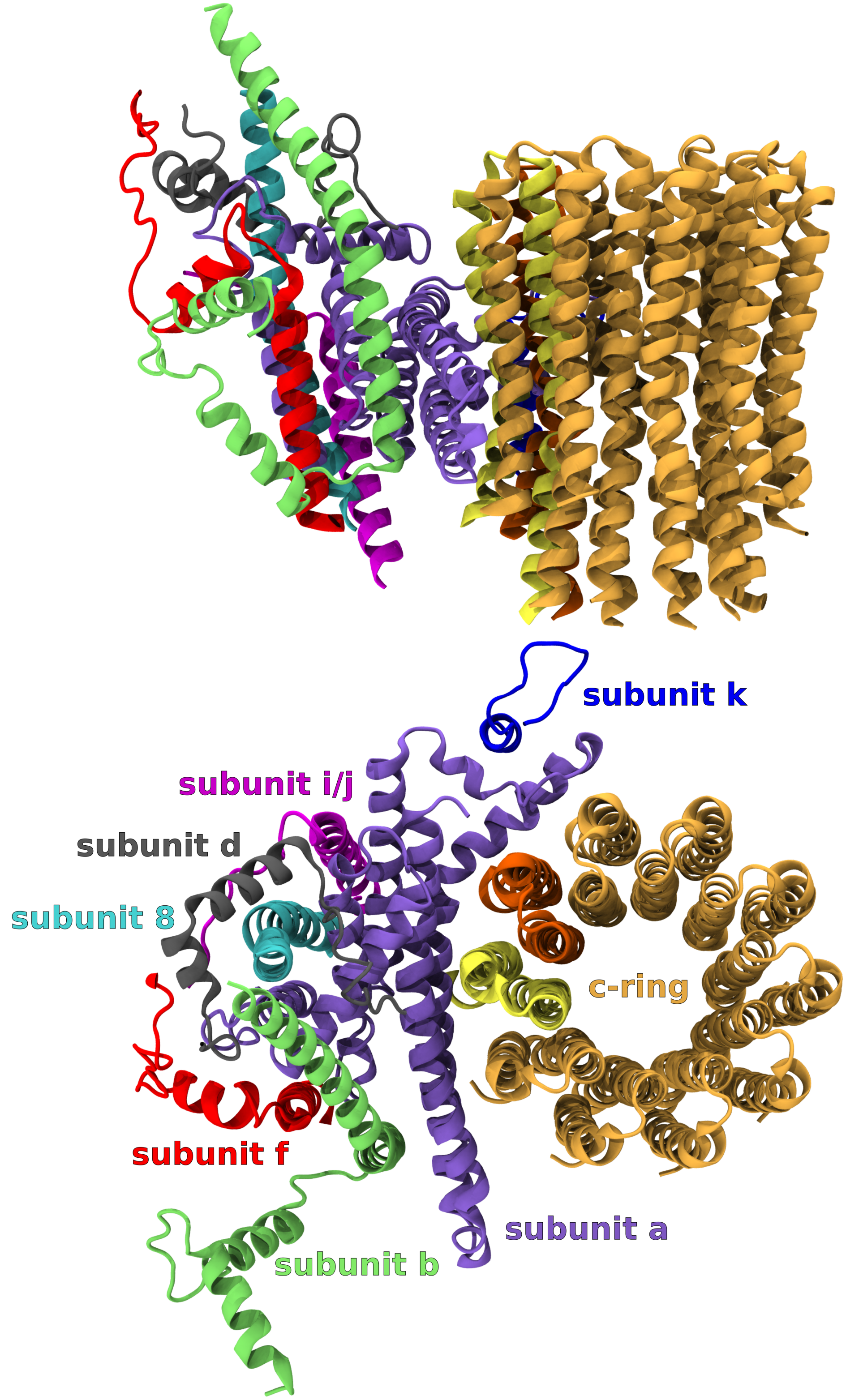

Figure S2: Subunit composition of the simulated F_o complex (top – side view, bottom – view from the mitochondrial matrix). Consistently with Fig. 1., the two c-subunits located in the proton-binding and proton-release half-channels are shown in yellow and red, respectively. The F_o complex was stable throughout the simulation, as reflected in RMSD over time for individual subunits (see Fig. S19).

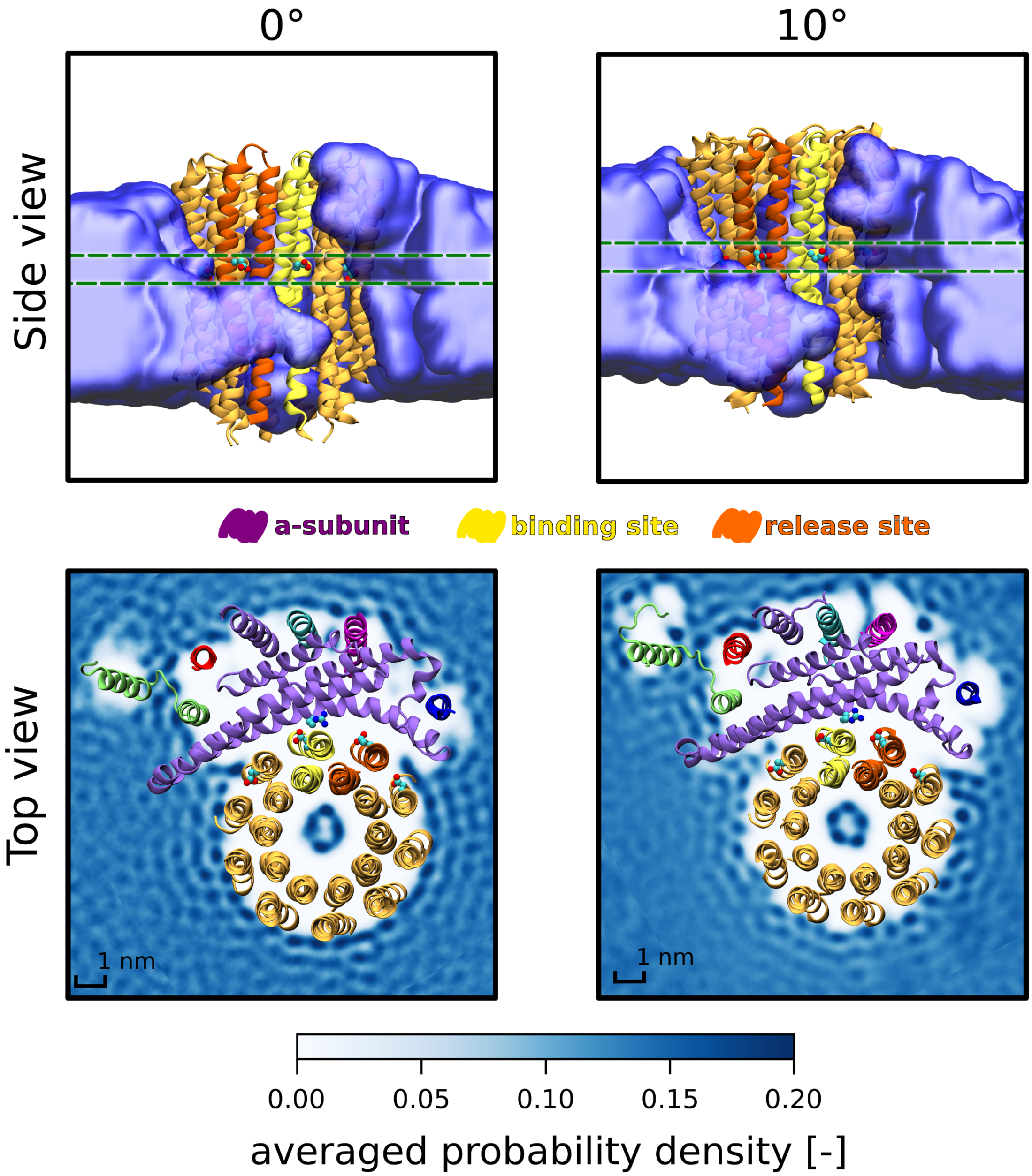

Figure S3: Lipid electron densities around the F_o complex with the c-ring at its initial position (0°, left) and rotated by 10° (right), averaged over 600 ns MD trajectories. The top view shows the density of the membrane hydrocarbon core at the average position of the proton-carrying glutamate residues (E59), indicated by the green dash lines in the side view. The side view panels show the selected electron density isosurfaces (0.1) from the perspective of the c-ring/a-subunit interface. The binding and release site c-subunits are shown in yellow and dark orange, respectively. As can be seen, lipids do not directly interact with the binding and release site glutamates, neither at 0° nor at 10°.

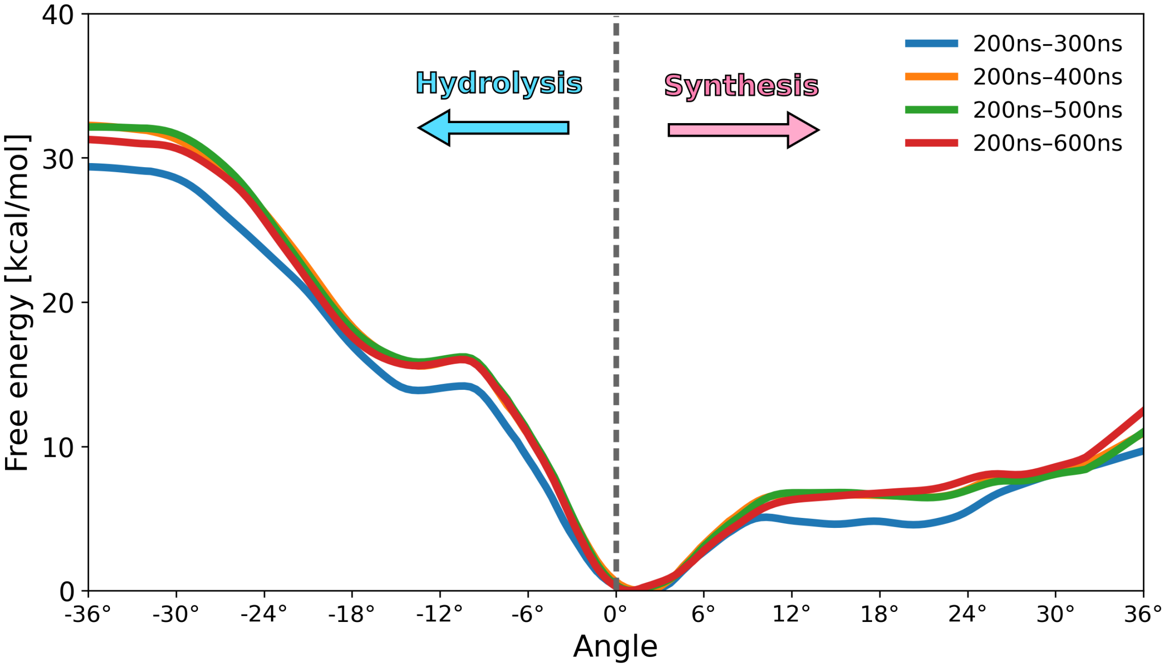

Figure S4: Convergence of the free energy profiles for the rotation of the wild type F_o (R^-B^0) in the synthesis and hydrolysis direction, with the length of umbrella sampling trajectories taken for analysis.

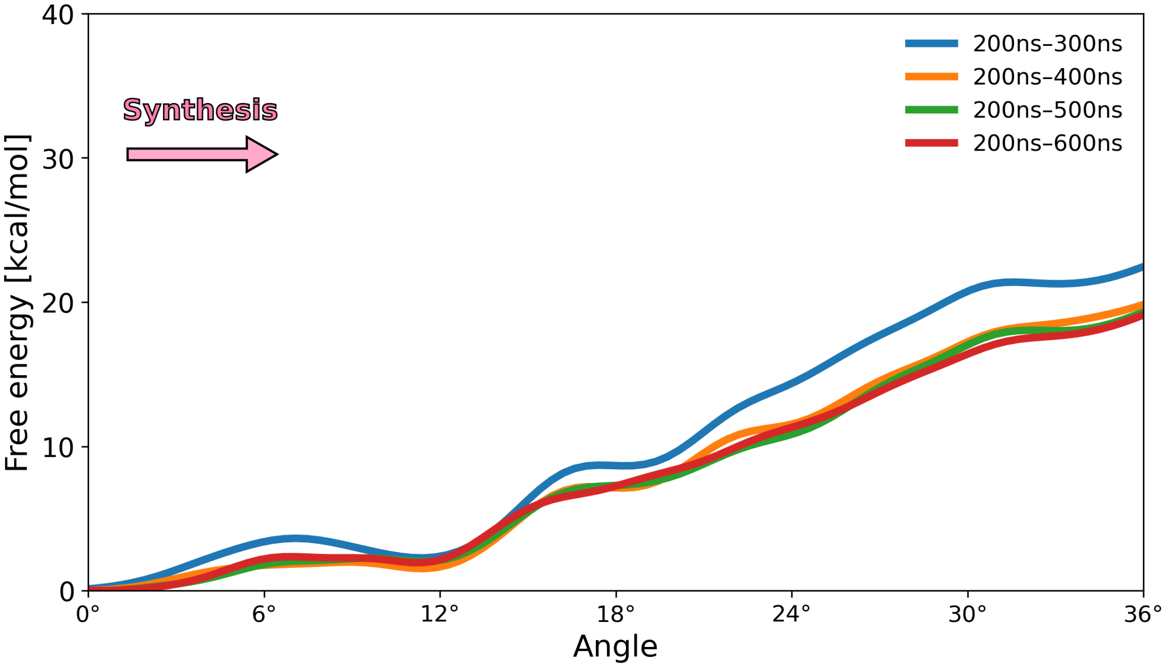

Figure S5: Convergence of the free energy profile for the rotation of the wild type F_o (R^-B^-) in the synthesis direction, with the length of umbrella sampling trajectories taken for analysis.

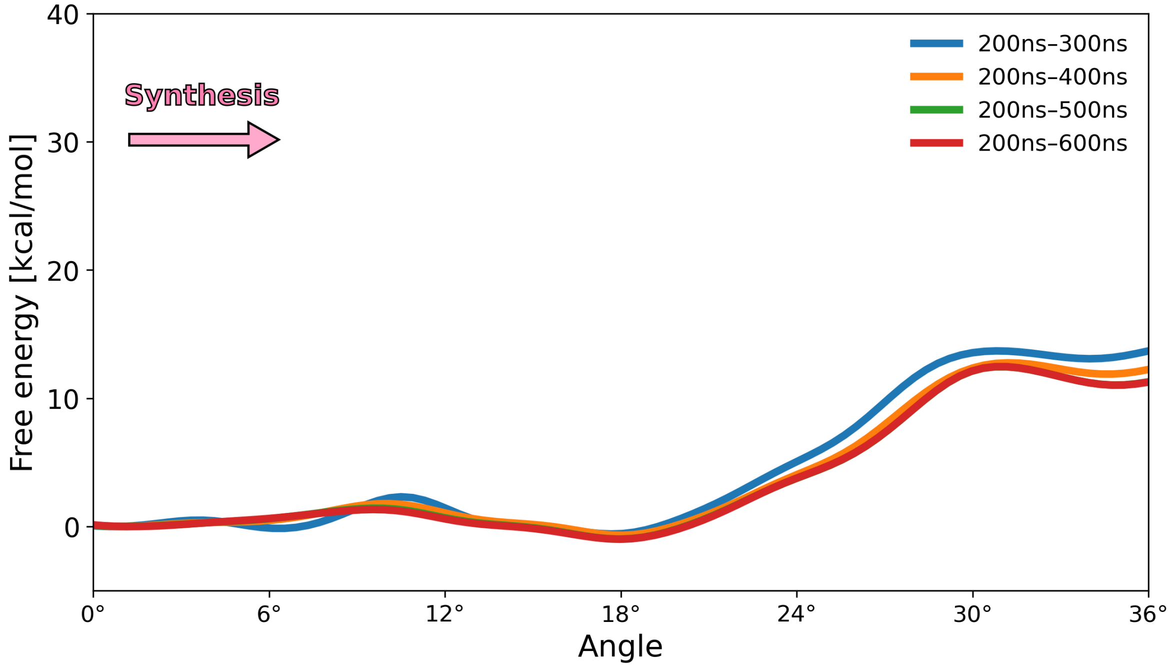

Figure S6: Convergence of the free energy profile for the rotation of the wild type F_o ((pre-R)$^-$R$^-$) in the synthesis direction, with the length of umbrella sampling trajectories taken for analysis.

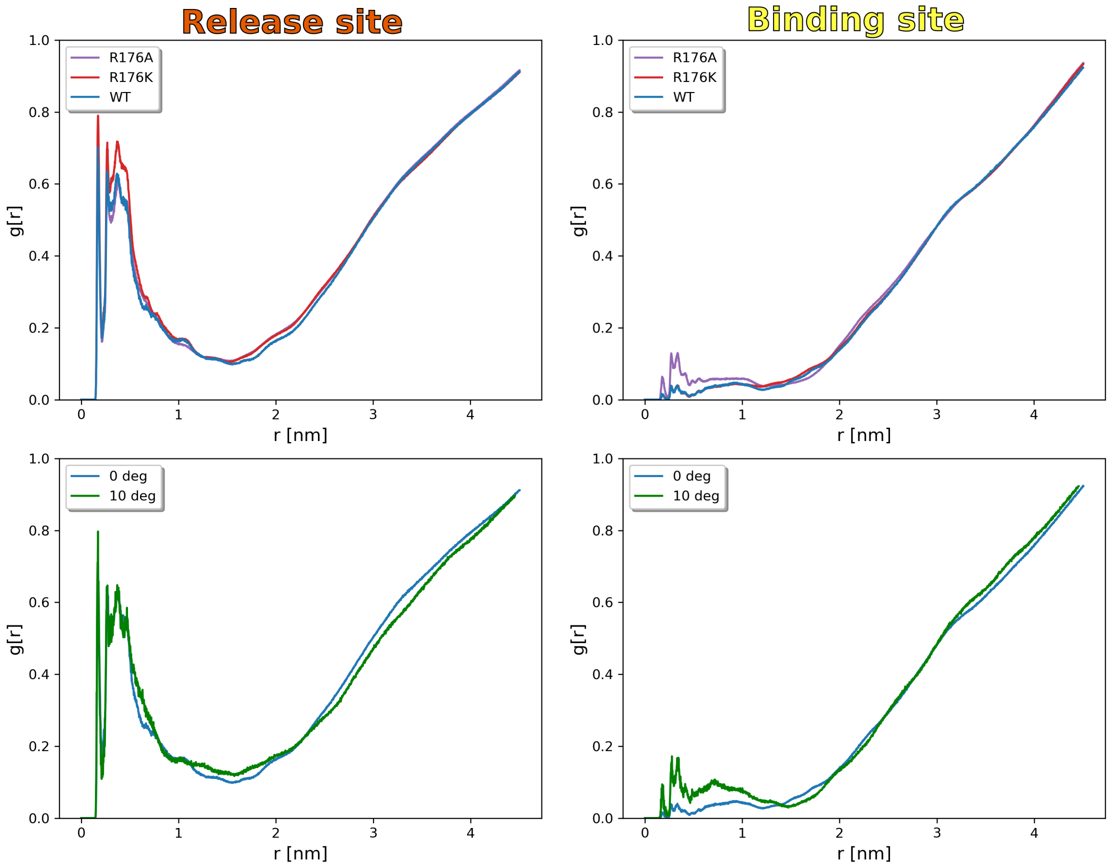

Figure S7: Radial distribution functions (RDF) for the water molecules (represented by their oxygen atom) around the heavy atoms of the carboxylate groups in the proton-release (left) and proton-binding (right) sites. (top) Dependence of RDFs on the type of side chain present at position 176, computed at the initial angular position of the c-ring (0°). (bottom) Comparison of the RDFs calculated for the wild-type F_o at 0° and at 10°.

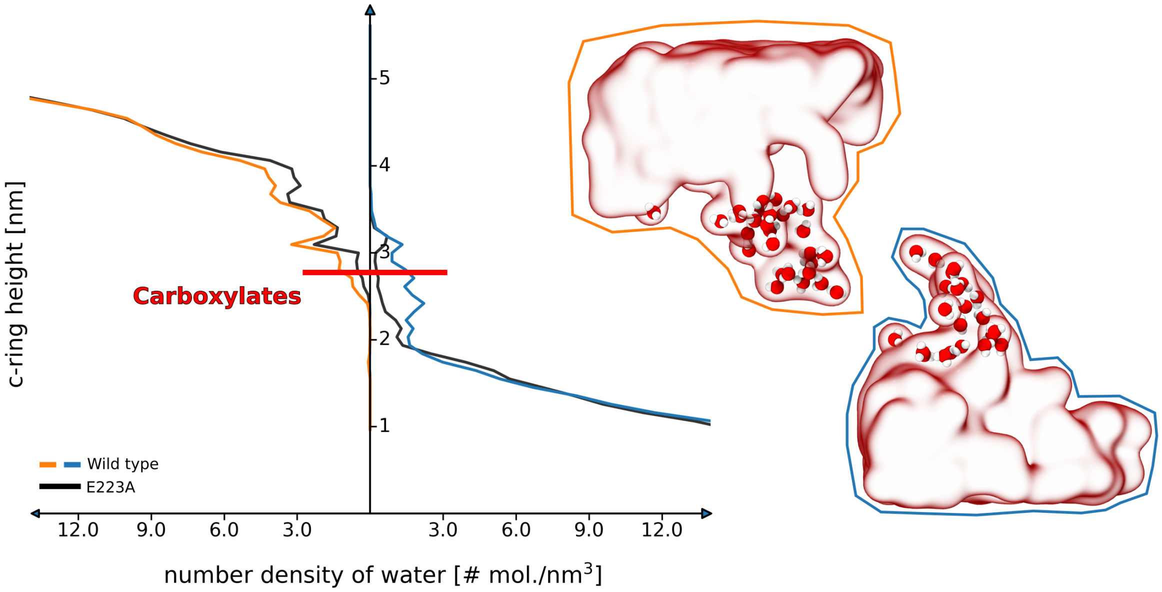

Figure S8: Number density of water in the proton-access half-channels as a function of the c-ring height for wild type system and E223A mutation. Density in the proton-binding and proton-release half-channel are shown in blue and orange, respectively, and corresponding densities in E223A mutant are shown in black. Red line indicates the height at which the c-ring carboxylates (B and R) are located.

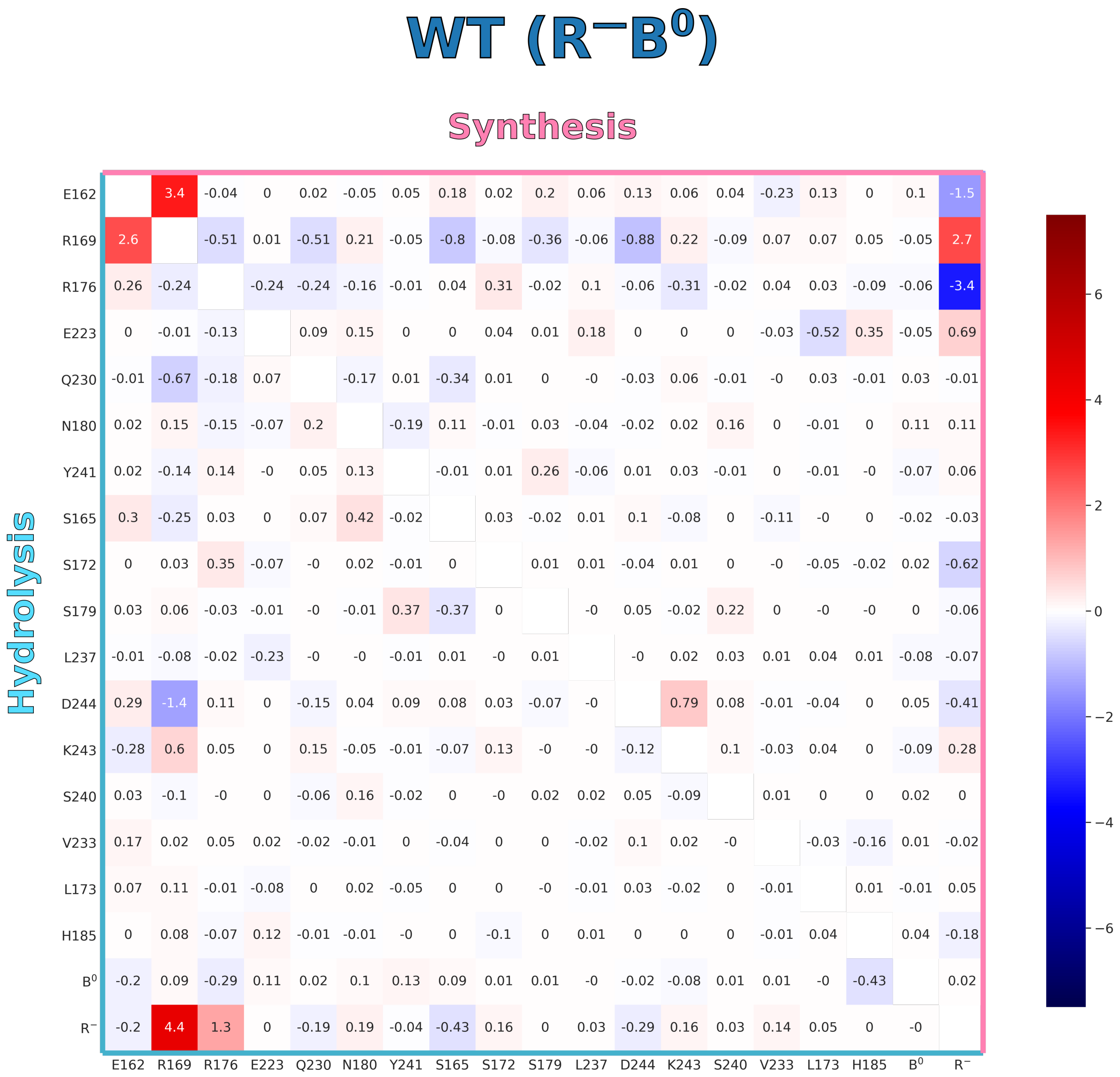

Figure S9: Average slopes (in kcal/(mol·deg)) of the selected pairwise interactions (electrostatic + vdW) as a function of the rotation angle, calculated in the 0–18° range. The values shown in the upper and lower triangle correspond to the rotation of the wild type F_o (R^-B^0) in the synthesis and hydrolysis direction, respectively. Locations of the residues used in the analysis are shown in Fig. S18.

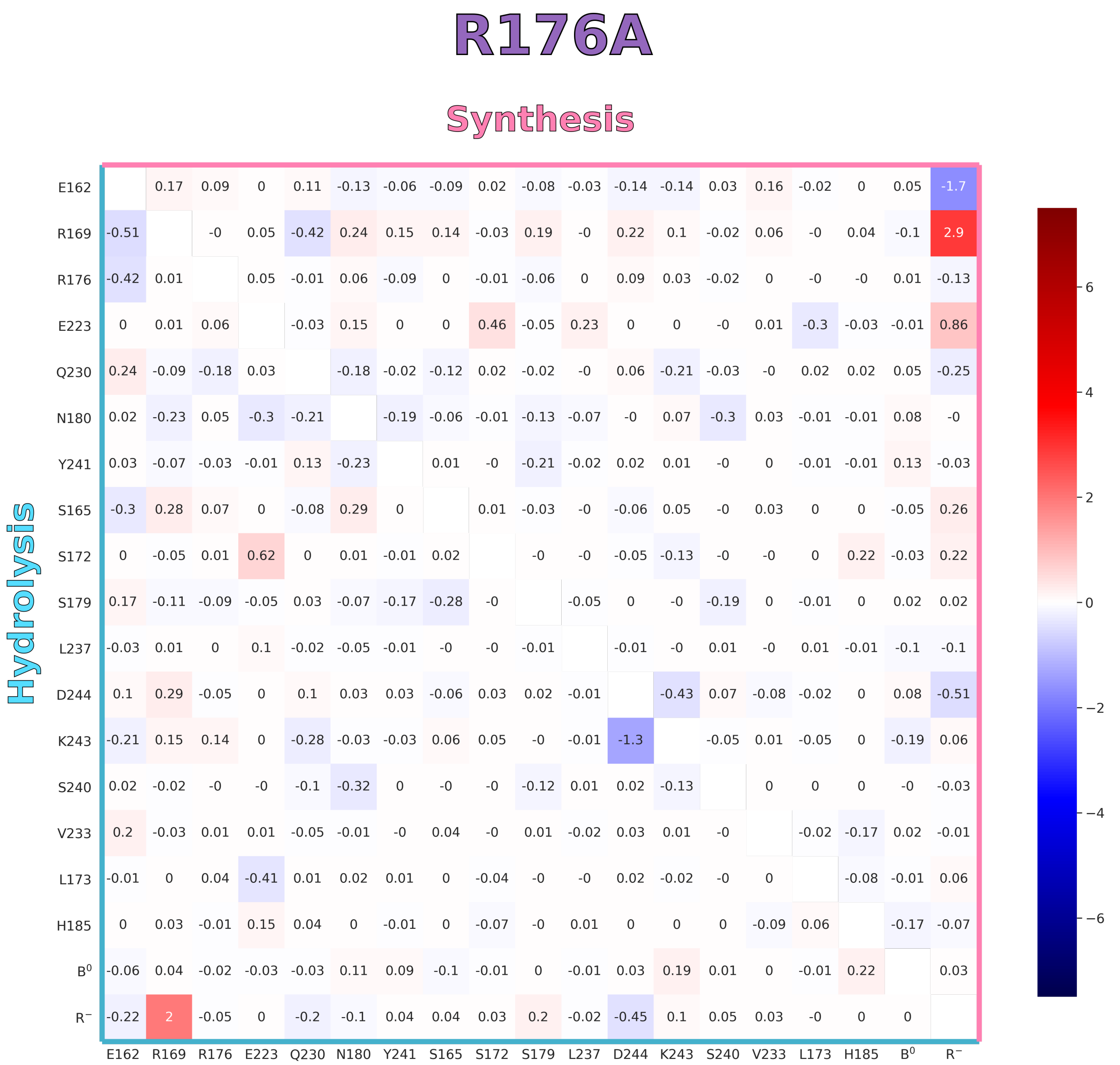

Figure S10: Average slopes (in kcal/(mol·deg)) of the selected pairwise interactions (electrostatic + vdW) as a function of the rotation angle, calculated in the 0–18° range. The values shown in the upper and lower triangle correspond to the rotation of the F_o R176A mutant in the synthesis and hydrolysis direction, respectively. Locations of the residues used in the analysis are shown in Fig. S18.

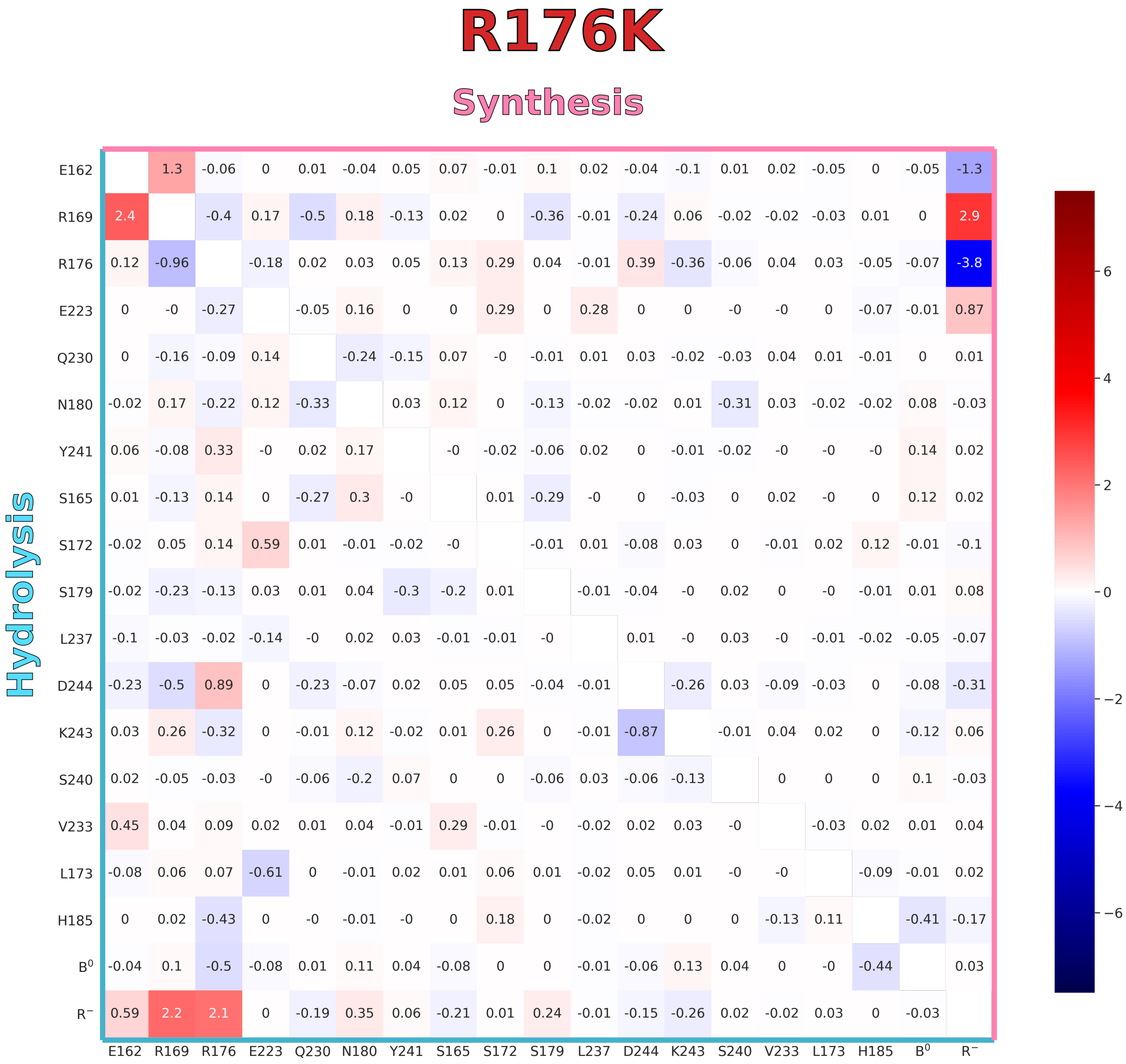

Figure S11: Average slopes (in kcal/(mol·deg)) of the selected pairwise interactions (electrostatic + vdW) as a function of the rotation angle, calculated in the 0–18° range. The values shown in the upper and lower triangle correspond to the rotation of the F_o R176K mutant in the synthesis and hydrolysis direction, respectively. Locations of the residues used in the analysis are shown in Fig. S18.

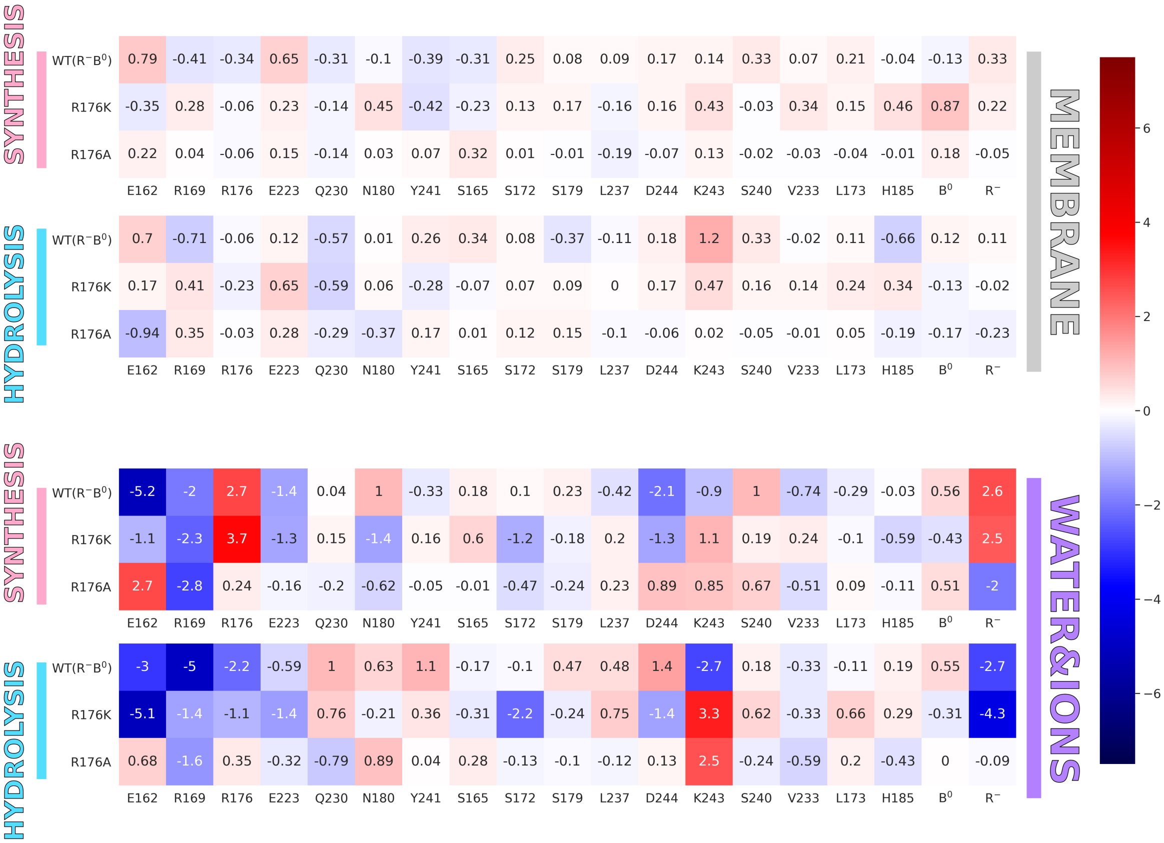

Figure S12: Average slopes (in kcal/(mol·deg)) of the sum of electrostatic and vdW interaction energies between the selected F_o residues and membrane lipids (MEMBRANE) or the water solution (WATER&IONS) as a function of the rotation angle, calculated in the 0–18° range. The values corresponding to the synthesis and hydrolysis direction are labeled accordingly. Three rows in each of the matrices correspond to wild type F_o (R^-B^0) and its R176K and R176A mutants. Locations of the residues used in the analysis are shown in Fig. S18.

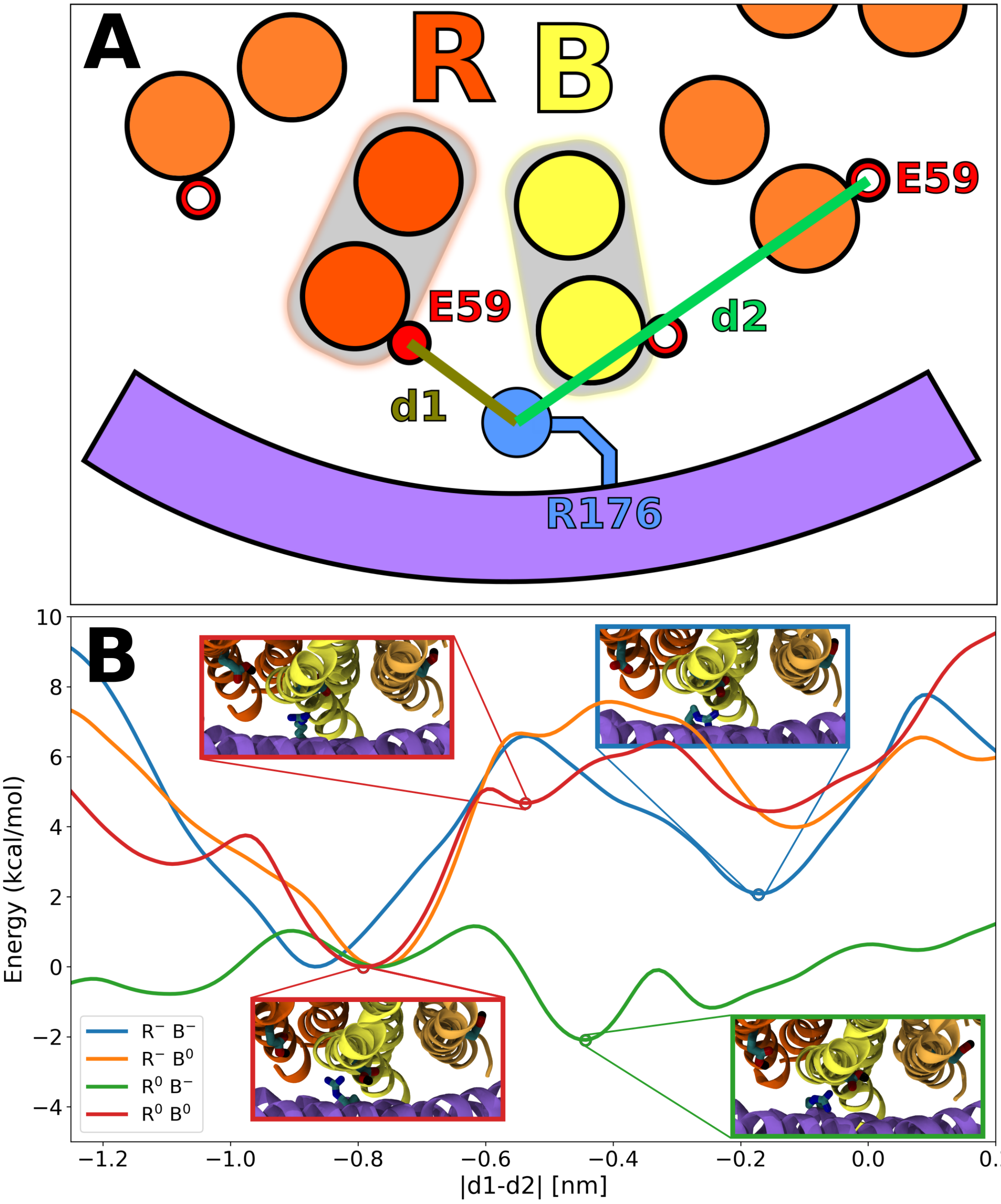

Figure S13: (A) Schematic of the setup of metadynamics simulations of the conformational bahavior of R176. By defining the reaction coordinate (rc) as the distance difference, |d1-d2|, we could examine the energetics of the flipping transition of R176 between the proton-binding half-channel (positive rc values) and the proton-release half-channel (negative rc values). (B) Free energy profiles for the flipping conformational transition of R176 between both half-channels at different protonation states of the B and R site carboxylates. In all but the highly unfavorable R^0B^- protonation state R176 shows a 2–4 kcal/mol preference for the release half-channel.

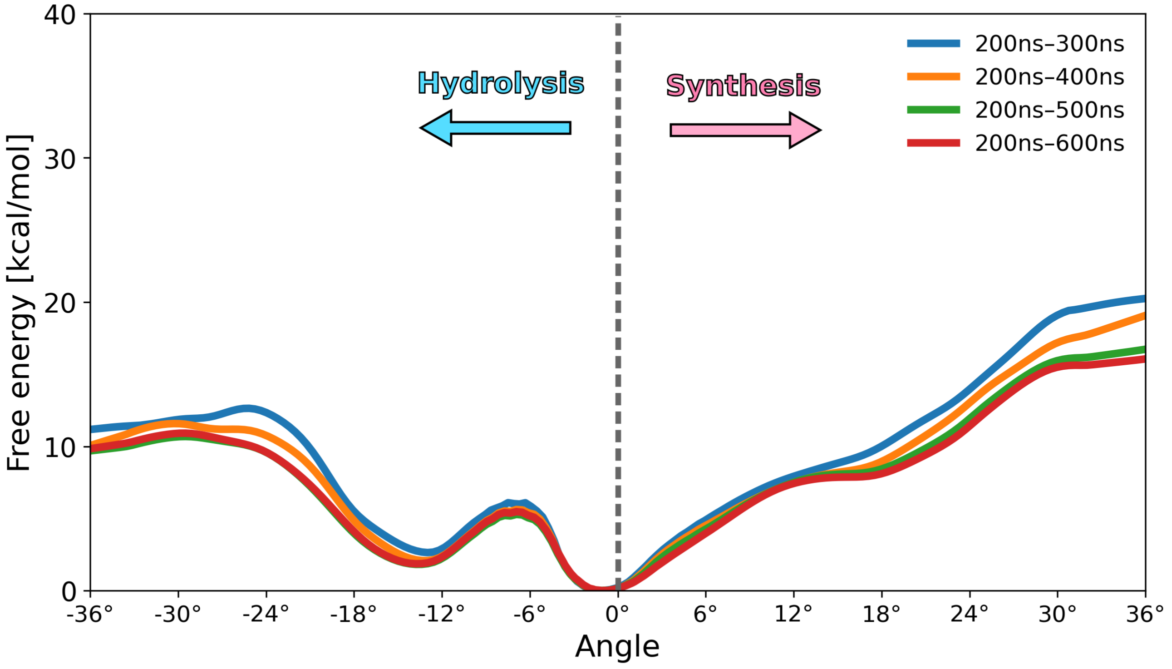

Figure S14: Convergence of the free energy profiles for the rotation of the R176A F_o mutant in the synthesis and hydrolysis direction, with the length of umbrella sampling trajectories taken for analysis.

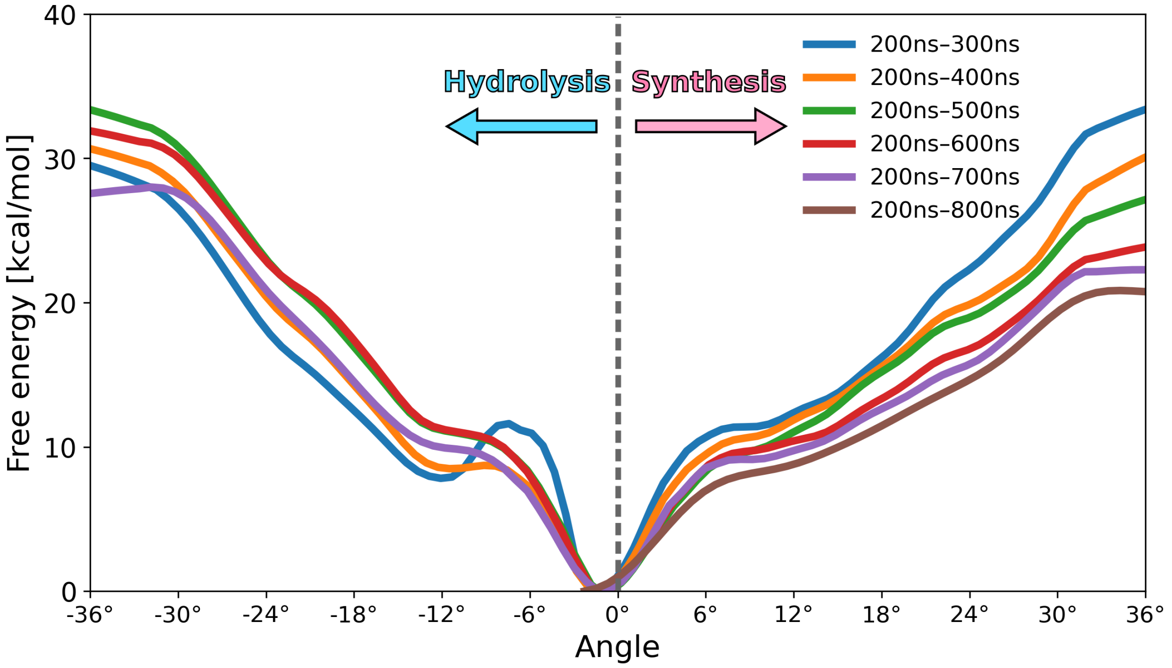

Figure S15: Convergence of the free energy profiles for the rotation of the R176K F_o mutant in the synthesis and hydrolysis direction, with the length of umbrella sampling trajectories taken for analysis.

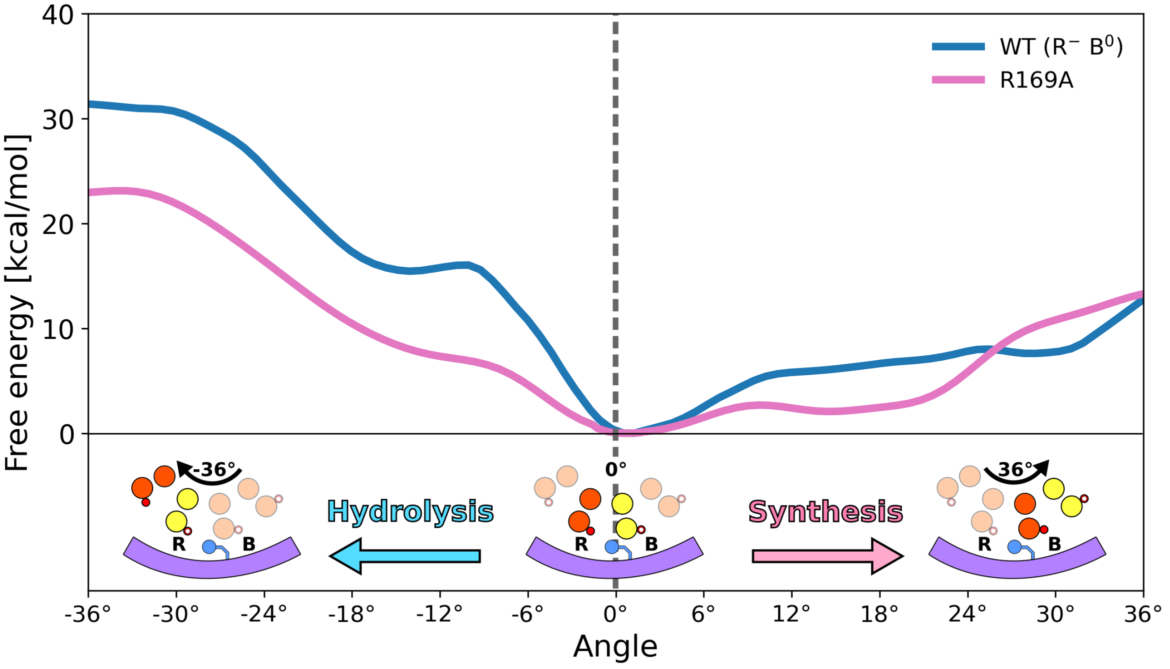

Figure S16: Response of the free energy profile for the F_o rotation to the mutation of the secondary arginine (R169) in the a-subunit to alanine.

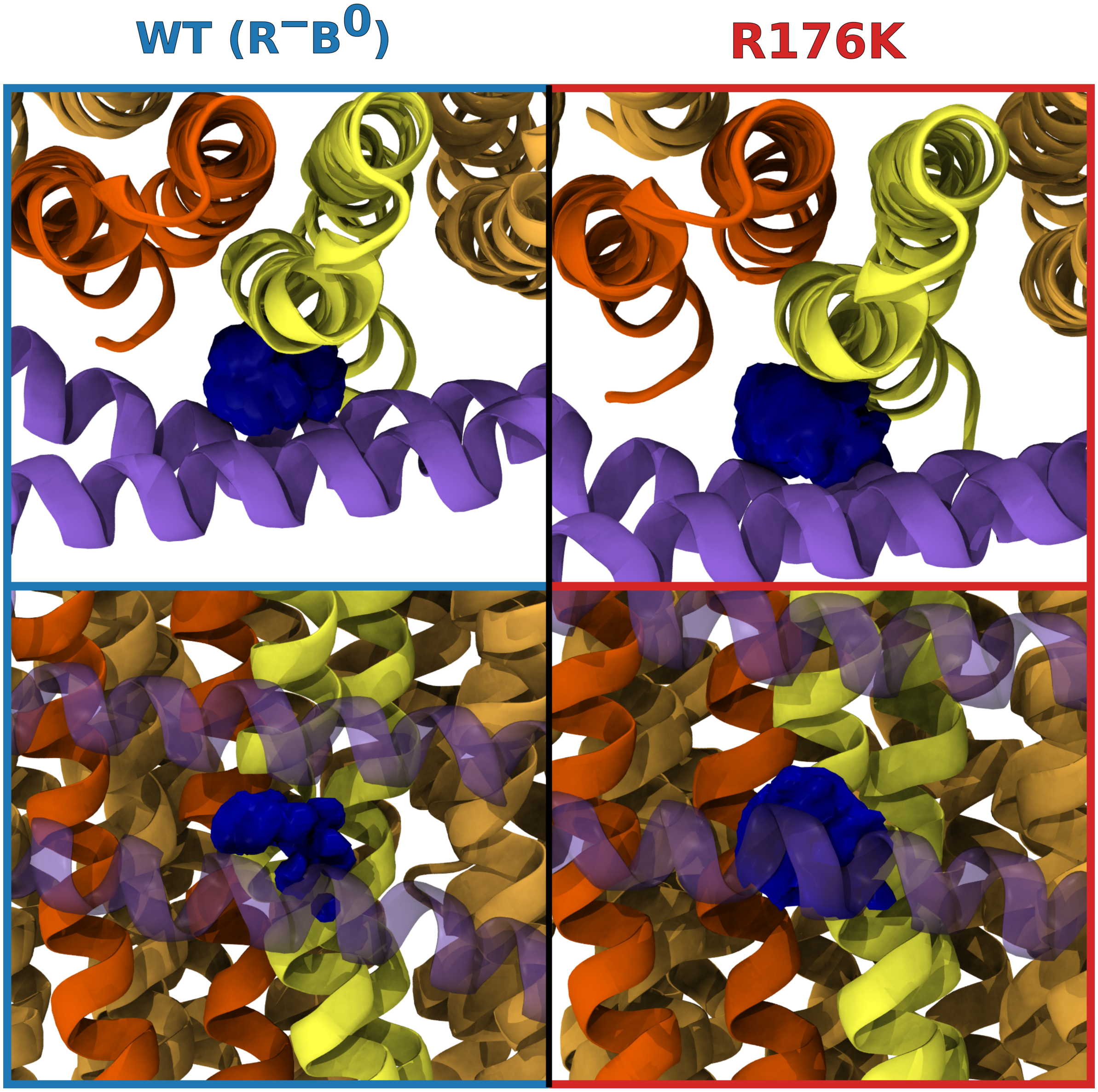

Figure S17: Top (upper panel) and side (lower panel) views of the side chain densities of R176 (WT) and K176 (R176K mutant) averaged over 1 μs unbiased simulations. The arginine adopts a single stable conformation, to the extent that its density reflects the sidechain shape. In contrast, the lysine sidechain is highly mobile such that its average density has a roughly spherical shape, meaning it samples multiple conformations at the c-ring/a-subunit interface, possibly accounting for the increased flux of water between the two half-channels.

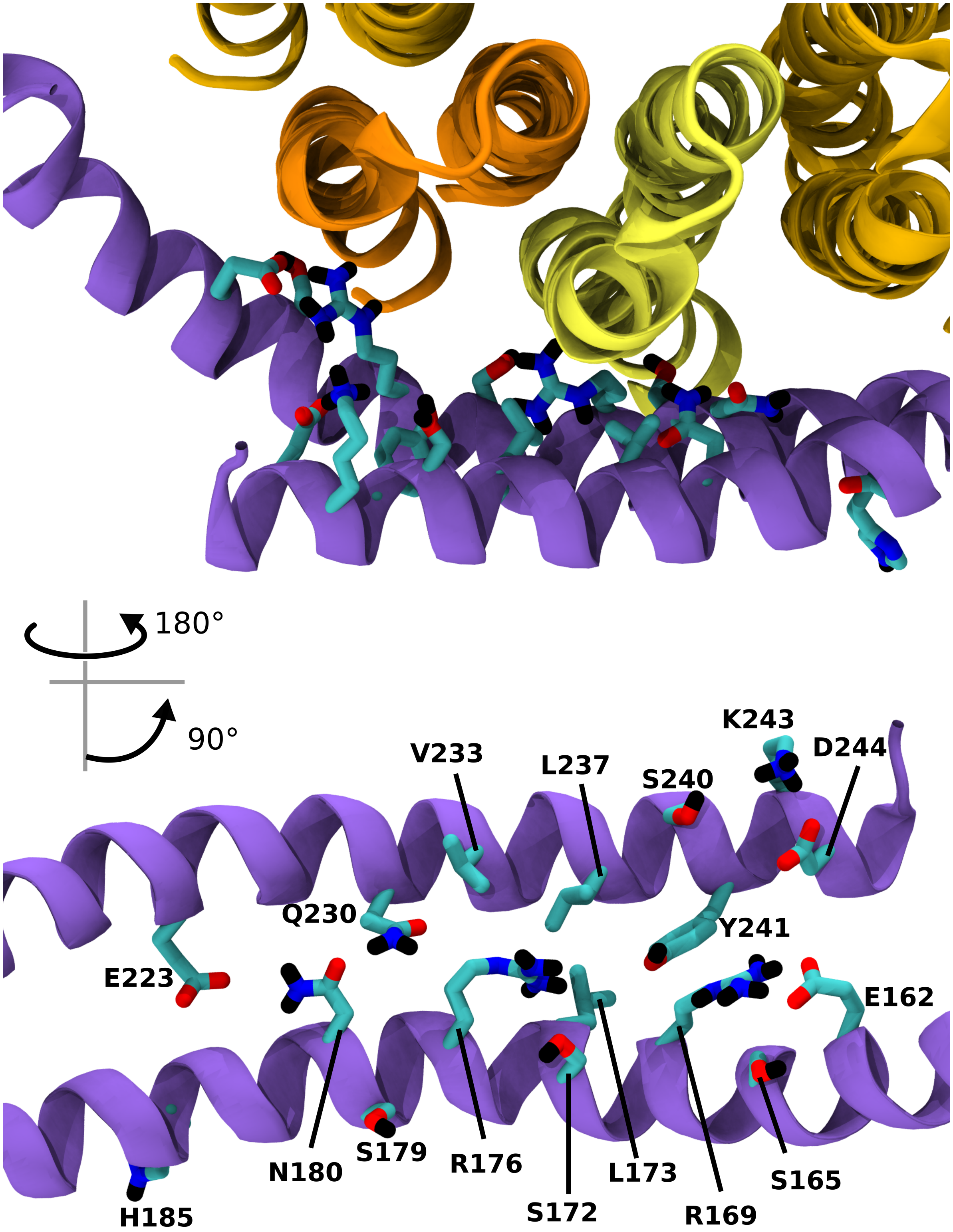

Figure S18: Residues in the a-subunit included explicitly in the analysis of pairwise enthalpic contributions to interaction free energies (Fig. S9, Fig. S10, Fig. S11 and Fig. S12). The selected residues are shown as seen from the mitochondrial matrix (top) and from the perspective of the c-ring (bottom).

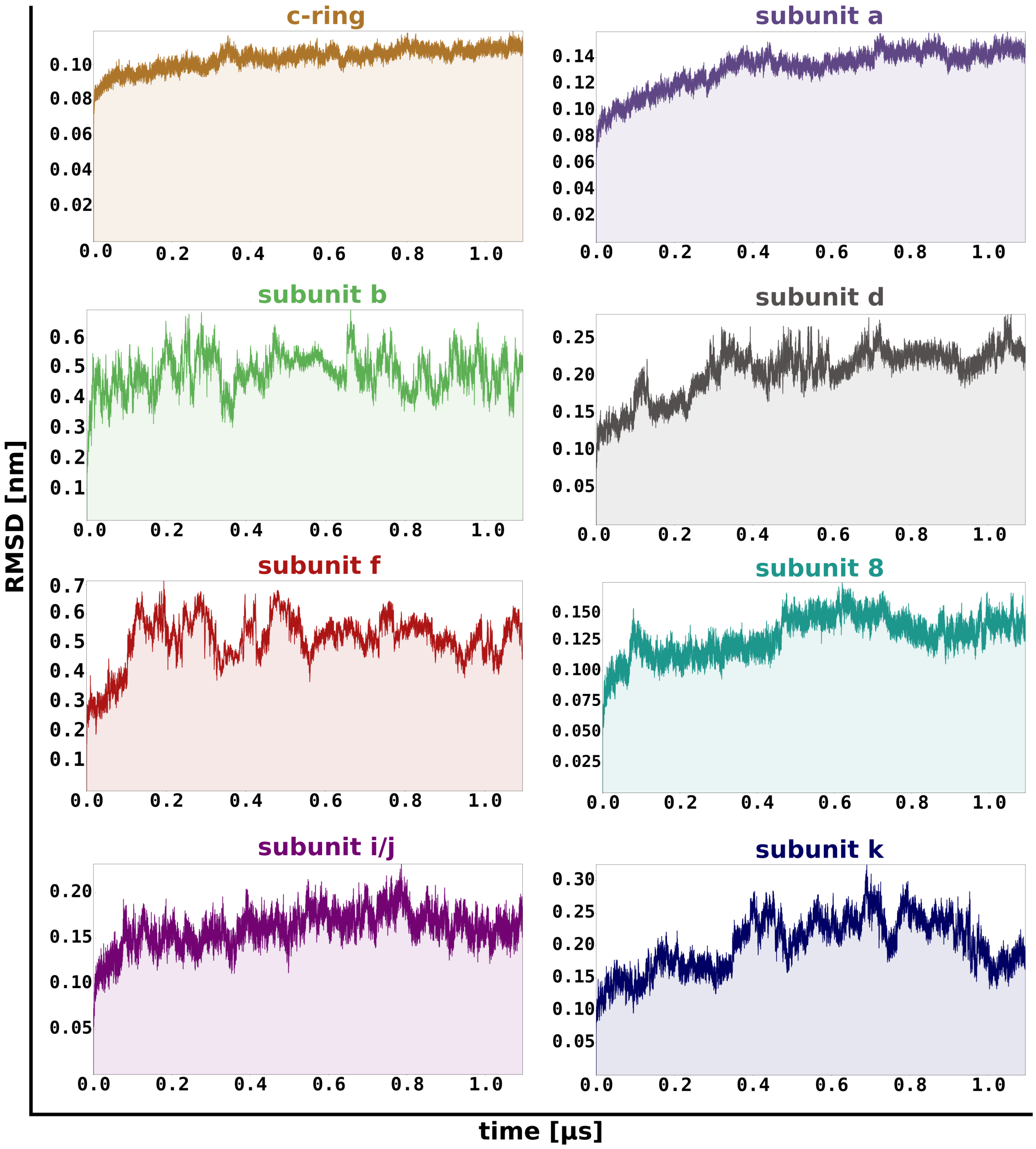

Figure S19: Root mean square deviation (RMSD) for the C_α atoms of each of the subunits comprising the simulated F_o complex as function of the simulation time (see Fig. S2). Since the conformationally flexible b-subunit and f-subunit undergo relaxation when embedded in a model of the inner mitochondrial membrane, their RMSDs reach plateau at significantly higher values than for the remaining more rigid subunits.

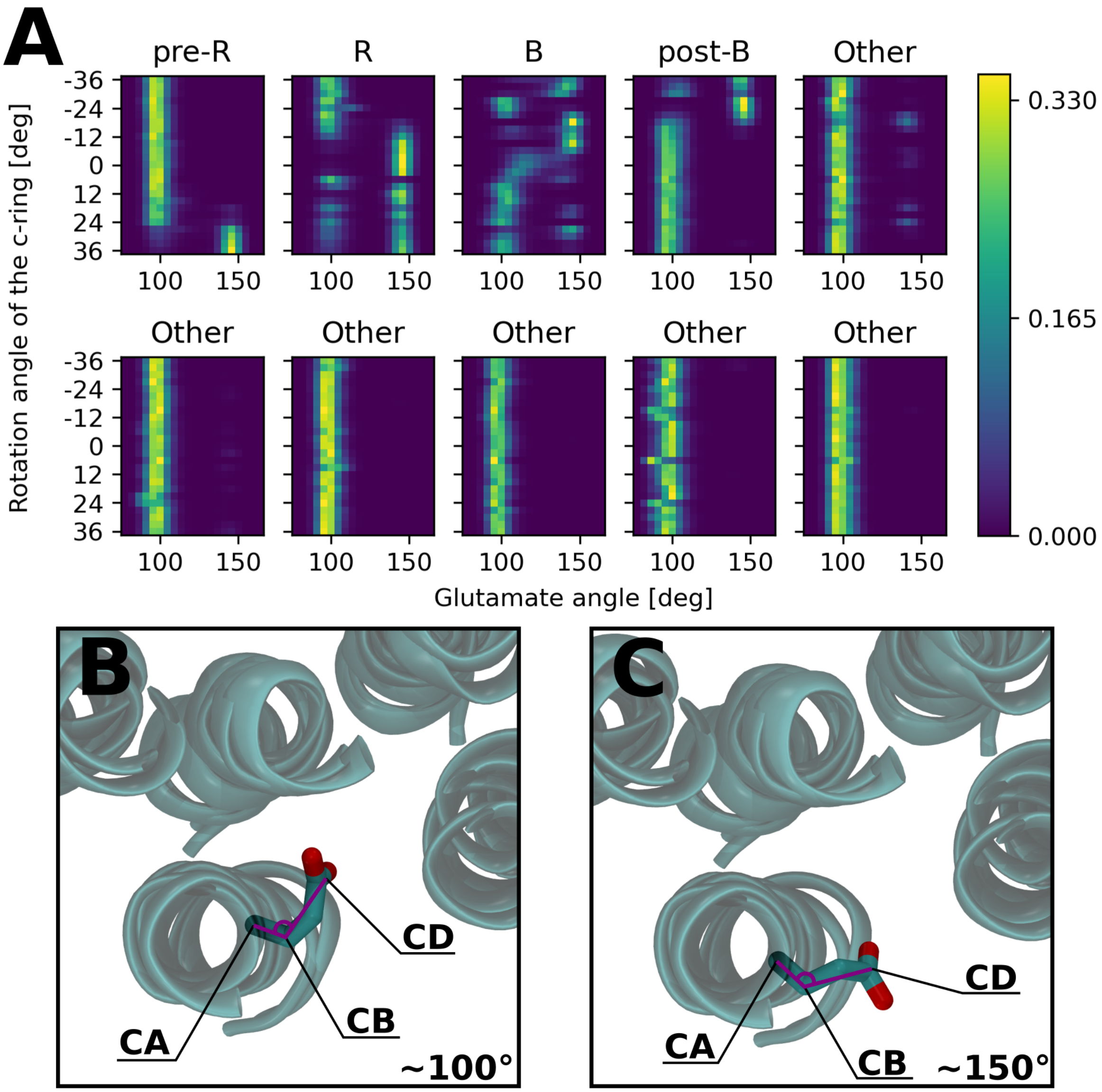

Figure S20: (A) Dependence of the conformation of the proton-carrying glutamates on the c-ring rotary angle. The conformation is defined by the angle CA–CB–CD and the color maps show how distributions of this angle depend on the c-ring rotary angle (glutamates are labeled in the same way as elswhere in the paper). As discussed previously in the literature,[1] the glutamates can assume two stable conformations: closed (100°), depicted in (B), and open (150°), depicted in (C). The closed conformation is clearly preferred by the protonated glutamates both at the B site and exposed to the lipid environment, while the open one is adopted by both protonated and unprotonated glutamates at the R site.
